# Cloning, Expression and Characterization of 3-Hydroxyisobutyrate Dehydrogenase from *Pseudomonas denitrificans* ATCC 13867

**DOI:** 10.1371/journal.pone.0062666

**Published:** 2013-05-01

**Authors:** Shengfang Zhou, Subramanian Mohan Raj, Somasundar Ashok, Selvakumar Edwardraja, Sun-gu Lee, Sunghoon Park

**Affiliations:** 1 Department of Chemical and Biomolecular Engineering, Pusan National University, Geumjeong-gu, Busan, Korea; 2 Centre for Research and Development, PRIST University, Thanjavur, India; 3 Technical University of Munich, Lehrstuhl für Biologische Chemie, Freising-Weihenstephan, Germany; La Trobe University, Australia

## Abstract

The gene encoding an NAD^+^-dependent, 3-hydroxyisobutyrate dehydrogenase (3HIBDH-IV) from *Pseudomonas denitrificans* ATCC 13867 was cloned and expressed in *Escherichia coli* BL 21 (DE3) and characterized to understand its physiological relevance in the degradation of 3-hydroxypropionic acid (3-HP). The deduced amino acid sequence showed high similarity to other 3-hydroxyisobutyrate dehydrogenase isozymes (3HIBDHs) of *P. denitrificans* ATCC 13867. A comparison of 3HIBDH-IV with its relevant enzymes along with molecular docking studies suggested that Lys171, Asn175 and Gly123 are important for its catalytic function on 3-hydroxyacids. The recombinant 3HIBDH-IV was purified to homogeneity utilizing a Ni-NTA-HP resin column in high yield. 3HIBDH-IV was very specific to (*S*)-3-hydroxyisobutyrate, but also catalyzed the oxidation of 3-HP to malonate semialdehyde. The specific activity and half-saturation constant (*K*_m_) for 3-HP at 30°C and pH 9.0 were determined to be 17 U/mg protein and 1.0 mM, respectively. Heavy metals, such as Ag^+^ and Hg^2+^, completely inhibited the 3HIBDH-IV activity, whereas dithiothreitol, 2-mercaptoethanol and ethylenediaminetetraacetic acid increased its activity 1.5–1.8-fold. This paper reports the characteristics of 3HIBDH-IV as well as its probable role in 3-HP degradation.

## Introduction

Several strains of *Escherichia coli* were recently developed to produce the commercially important chemical, 3-hydroxypropionic acid (3-HP), from glycerol [1], [2]. Although the final titer was high (∼39 g/L), the requirement of an exogenous supply of high-cost coenzyme B_12_ by one essential enzyme, glycerol dehydratase, was the major obstacle to the use of *E. coli* in the commercial production of 3-HP. One strategy to overcome this limitation is to construct a 3-HP synthetic route in a host microorganism, such as *Pseudomonas denitrificans*, which can naturally synthesize coenzyme B_12_ under aerobic conditions [3]. On the other hand, when the production of 3-HP in genetically modified *P. denitrificans* was attempted, the 3-HP produced was degraded in the late exponential growth phase (unpublished results).

The degradation of 3-HP in *P. denitrificans* was attributed to the existence of 3-HP degradative enzymes and pathways. According to the literature, 3-HP can be degraded via two routes: 1) malonate semialdehyde, which is generated by 3-hydroxypropionate dehydrogenase (EC: 1.1.1.59), and 2) 3-hydroxypropionyl-CoA, which is produced by either 3-hydroxypropionyl-CoA synthetase (EC: 6.2.1.36) or 3-hydroxyisobutyryl-CoA hydrolase (EC: 3.1.2.4) (www.keggpathway.com) [4]–[7]. Malonate semialdehyde is further converted to acetyl-CoA and enters the main stream of the energy metabolism, whereas 3-hydroxypropionyl-CoA is converted to acryloyl-CoA and enters amino acid metabolism [4], [5]. In *P. denitrificans*, however, 3-HP degradation and enzymes, such as 3-HP dehydrogenase and 3-hydroxypropionyl-CoA synthetase or 3-hydroxyisobutyryl-CoA hydrolase, have not been reported.

In an effort to develop an efficient *P. denitrificans* for coenzyme B_12_-free 3-HP production, we examined the enzymatic degradation of 3-HP. Because 3-HP dehydrogenase has not been identified in *P. denitrificans* and other *Pseudomonas* species, a similar but relatively well studied enzyme, 3-hydroxyisobutyrate dehydrogenase (3HIBDH), was chosen and examined for 3-HP degradation. 3HIBDH catalyzes the reversible oxidation of 3-hydroxyisobutyrate (3-HIB, C_4_H_7_O_3_) to methylmalonate semialdehyde (C_4_H_6_O_3_), which is an intermediary metabolite of valine degradation. Furthermore, a few 3HIBDHs were reported to oxidize several 3-hydroxyacids, including 3-HP [7]–[9]. *P. denitrificans* has four putative isozymes of 3-HIBDHs, namely 3HIBDH-I, II, III and IV, according to a search using the gene encoding 3HIBDH of *P. fluorescens* Pf5 ATCC BAA-477 (accession no., AAY90588) [10]. Among these isozymes 3HIBDH-IV was chosen for detailed investigation in this study, because it was expected to play a major role in 3-HP degradation. 3HIBDH-IV was cloned, expressed and purified from the recombinant strain *E. coli* 3HIBDH4 and its kinetic characteristics on a range of substrates, including 3-HP, were examined. The probable role of 3HIBDH-IV in 3-HP degradation is also discussed.

## Materials and Methods

### Materials

The genomic DNA isolation kit and pGEM-T vector were purchased from Promega (Madison, WI, USA). The high-fidelity DNA polymerase was obtained from Invitrogen (Seoul, Korea). The primers were synthesized by Cosmotech Co. Ltd., Korea. The restriction and DNA-modifying enzymes were supplied by New England Bio-labs (Beverly, MA, USA). The pQE-80L vector, miniprep and DNA gel purification kits were purchased from Qiagen (Mannheim, Germany). The Ni-NTA-HP resin column was obtained from GE Healthcare (Sweden). 3-HP was acquired from Tokyo Kasei Kogyo Co. Ltd., Japan (TCI America). Unless otherwise indicated, all other chemicals, cofactors and enzymes were supplied by Sigma-Aldrich (St. Louis, MO, USA).

### Real-time PCR for Quantification of mRNA

*P. denitrificans* ATCC 13867 was grown in M9 medium with supplementation of 30 mM 3-HP and without supplementation of 3-HP, both under aerobic condition at 37°C and 200 rpm in an orbital incubator shaker. One milliliter of culture, contained 2×10^8^ cells, was collected during exponential growth phase into the vials containing two volumes of RNA protect reagent (Qiagen, Inc., USA). The culture mix was centrifuged at 5000×g for 10 min. Total RNA was isolated using RNA isolation kit (Qiagen, Inc., USA). Four microgram of total RNA was synthesized into first strand cDNA in a 20 µL reaction using SuperScript III first-strand synthesis system (Invitrogen, USA). Real-time PCR was performed according to SYBR green method [11] in a 20 µL reaction volume using Real-Time PCR system 7300 (Applied Biosystems, USA) under the following thermal cycling: predenaturing at 95°C for 30 s, followed by 40 cycles of 95°C for 5 s, 62°C for 30 s, and 72°C for 30 s. The reaction mixture contained 200 ng of cDNA, 10 µL of 2x SYBR *premix Ex Taq* (TaKaRa, Bio. Inc., Japan), 0.4 µL of 50x ROX reference dye (TaKaRa, Bio. Inc., Japan) and 10 pmol of forward and reverse primers corresponding to the genes *3hibdhI*, *3hibdhII*, *3hibdhIII*, and *3hibdhIV*. The primers used for amplification of *3hibdh*s are listed in Table 1. The *rpoD* gene, which encodes sigma factor 70 was used as reference gene. PCR efficiencies of all primers were experimentally determined and found to be suitable for reliable copy number quantification. Relative mRNA amounts were determined by the ΔΔCT method as described previously [12].

**Table 1:** Bacterial strains, plasmids and primers used in this study.

Strains and plasmids	Description	Source
Strains		
* P. denitrificans* ATCC13867	Source for 3*hibdhIV* gene	KCCM, Korea
* E. coli* XL-1 blue	Cloning host	KCCM, Korea
* E. coli* BL21 (DE3)	Expression host	Novagen
* E. coli* 3HIBDH4	*E. coli* BL21 (DE3) harboring *3hibdhIV* gene	This study
* E. coli* 3HIBDH3	*E. coli* BL21 (DE3) harboring *3hibdhIII* gene	This study
* E. coli* 3HIBDH2	*E. coli* BL21 (DE3) harboring *3hibdhII* gene	This study
* E. coli* 3HIBDH1	*E. coli* BL21 (DE3) harboring *3hibdhI* gene	This study
Plasmids		
pGEM-T	*lacZα*; cloning vector; pGEM 5zf(+) derivative; 3′T-overhang; Amp^r^	Promega
pQE-80L	*lac*I^q^; expression vector; ColE1-*ori*; His_6_-N; Amp^r^	Qiagen
pT3HIBDH4	*3hibdhIV* gene in pGEM-T; Amp^r^	This study
pT3HIBDH3	*3hibdhIII* gene in pGEM-T; Amp^r^	This study
pT3HIBDH2	*3hibdhII* gene in pGEM-T; Amp^r^	This study
pT3HIBDH1	*3hibdhI* gene in pGEM-T; Amp^r^	This study
pQ3HIBDH4	*3hibdhIV* gene in pQE-80L; Amp^r^	This study
pQ3HIBDH3	*3hibdhIII* gene in pQE-80L; Amp^r^	This study
pQ3HIBDH2	*3hibdhII* gene in pQE-80L; Amp^r^	This study
pQ3HIBDH1	*3hibdhI* gene in pQE-80L; Amp^r^	This study
Primers used for RT PCR (forward, F; Reverse, R)	Sequence 5′ to 3′	
* 3hibdhI*, F	TGC TGG AGT GCT CCA CCA T	This study
* 3hibdhI*, R	GAC CAT GAA GGT CAG GGT	This study
* 3hibdhII*, F	ATG TCT GCT GCC TTG CCT TCC ATT GC	This study
* 3hibdhII*, R	AGG CAC AGC ATC ACC ACT T	This study
* 3hibdhIII*, F	ATG GCA AAA GTC GCT TTC ATC G	This study
* 3hibdhIII*, R	CGA ACT GAT CGA CCC ATT	This study
* 3hibdhIV*, F	GTG ATC ATC ACC ATG CTG CCT	This study
* 3hibdhIV*, R	AGC ATT CAG CGG GTC GAT GGT G	This study
* rpoD*, F	GAA GTC GGC AAG CAG TTC GAT G	This study
* rpoD*, R	TCA CTC GTC GAG GAA GGA GCG CA	This study

### Cloning of 3*hibdhIV*

Table 1 lists the bacterial strains and plasmids used in this study. *E. coli* BL21 (DE3) served as a host for developing the strain, whereas *E. coli* XL1-blue was used for routine cloning and plasmid maintenance. The LB medium was used for the routine culture and growth of *E. coli*. Ampicillin at 100 mg/L was added to the culture media. Gene manipulations were carried out using standard methods [13]. The plasmid pQE-80L was used to clone the 3*hibdhIV* gene in *E. coli* BL21 (DE3). The 5′ and 3′ terminal DNA sequences of the 3*hibdhIV* gene of *P. denitrificans* ATCC 13867 were used to design the following primers: upstream, 5′TTAGGATCCATGCGCATCGGTTTCATCGGACTCGGCAACATG-3′, and downstream, 5′- TACAAGCTTTCAGCCCTGCTCGTACAGCTTCACAATGGCTGAG-3′ (the underlined nucleotides indicate *Bam*HI and *Hin*dIII sites, respectively). These primers were used to amplify the coding region of the 3*hibdhIV* gene from the genomic DNA of *P. denitrificans* by PCR. The amplified PCR fragment was ligated into the pGEM-T vector and transformed into *E. coli* XL1-blue. The resulting plasmid, pT3HIBDH4, was sequenced by Cosmotech Co., Ltd., Korea. The pT3HIBDH4 plasmid was then digested with the *Bam*HI and *Hin*dIII restriction enzymes, and the restriction fragment was sub-cloned into the pQE-80L expression vector. The resulting expression plasmid pQ3HIBDH4 containing the 3*hibdhIV* gene with the His-tag at the *N*-terminus was transformed into *E. coli* BL21 to yield *E. coli* (pQ3HIBDH4), which is referred to as the SH-3HIBDH4 strain.

### Expression and Purification of Recombinant 3HIBDH-IV

The SH-3HIBDH4 strain was grown in LB medium supplemented with 100 mg/L ampicillin. The cells were grown aerobically in 1 L Erlenmeyer flasks containing 350 mL of medium at 30°C and 200 rpm in an orbital incubator shaker. The cells were induced at ∼0.6 OD_600_ with 0.5 mM isopropyl-beta-D-thiogalactopyranoside (IPTG) and incubated at 25°C for 10 h. The cells were then harvested and centrifuged at 10,000×g for 10 min. The pellet was washed twice with 50 mM potassium phosphate buffer (pH 8.0) and resuspended in the binding buffer (20 mM sodium phosphate buffer containing 0.5 M NaCl and 20 mM imidazole). The resuspended cells were disrupted using a French Pressure Cell (FA-078A, Thermo Electron Corp.; Waltham, MA, USA) at 1,250 psi. The cell lysate was centrifuged at 25,000×g at 4°C for 30 min to remove the particulate fraction. The soluble fraction was subjected to purification under non-denaturing conditions by Ni-affinity chromatography using a Ni-NTA-HP resin column (17-5248-01; GE Healthcare, Sweden). The eluents from the column was pooled and dialyzed using a 10 kDa cutoff membrane to remove the salts. The resulting enzyme extract was electrophoresed under denaturing conditions, as described by Laemmli [14], and the extract was stored at −80°C.

### Determination and Characterization of 3HIBDH-IV Activity

The 3HIBDH-IV activity was measured using the method described by Rougraff et al. [15]. The reaction mixture containing 100 mM Tris-HCl buffer (pH 9.0), 0.2 µg/mL enzyme and 3 mM 3-HP was incubated at 30°C for 5 min. The reaction was initiated by adding 2 mM NAD(P)^+^. The enzyme activity was determined by measuring the reduction of NAD(P)^+^ to NAD(P)H at 340 nm. The amount of NAD(P)H formed was determined using a molar extinction coefficient (Δε340) of 6.22×10^3^ M^−1^ cm^−1^. One unit of 3HIBDH-IV activity was defined as the amount of enzyme needed to reduce 1 µmol of NAD(P)^+^ to NAD(P)H in one minute. All enzyme activities were determined in triplicate and the values indicated are the triplicate measurement of the same enzyme preparation (mean values, n = 3).

The effects of temperature and pH on the 3HIBDH-IV activity were determined using 3-HP as a substrate and NAD^+^ as a cofactor. The temperature effects were examined at pH 9.0 in the range of 25 to 50°C using a temperature-controlled double beam spectrophotometer (Lambda 20, PerkinElmer; Norwalk, CT, USA). The pH effects were determined in the range of pH 6.0 to 10 at 30°C using 50 mM potassium phosphate (pH 6−7), Tris-HCl (pH 7−9) and Glycine-NaOH buffers (pH 9−10.6). The effects of metal ions and other chemicals on the 3HIBDH-IV activity were examined at 30°C and pH 9.0 using 3-HP and NAD^+^ as a substrate and cofactor, respectively. The effect of positive monovalent light alkali metals (Na^+^, Li^+^, K^+^ and NH_4_^+^), a positive bivalent alkali-earth metal (Mg^2+^), transition metals (Co^2+^, Ni^2+^, Cu^2+^, Zn^2+^, Fe^2+^ and Mn^2+^) and heavy metals (Hg^2+^ and Ag^+^), all in the form of chloride-sulfate salts, on the 3HIBDH-IV activities were evaluated at a 1 mM concentration. The effects of two disulfide reductants, such as 2-mercaptoethanol (2-ME) and dithiothreitol (DTT), and the chelating agent ethylenediaminetetraacetic acid (EDTA) were also evaluated at 1.0 mM. The substrate specificity of 3HIBDH-IV was determined at 30°C and pH 9.0 for many different substrates in the presence of NAD^+^ or NADP^+^ as cofactors. 3-hydroxypropionate, sodium (*R*)-3-hydroxyisobutyrate, sodium (*S*)-3-hydroxyisobutyrate, _L_-serine, α-methyl-_D_,_L_-serine, methyl-(*S*)-(+)-3-hydroxyl-2-methylpropionate, _D_-glycerate calcium salt dehydrate and methyl-2,2-dimethyl-3-hydroxypropionate, were used as the substrates in this study. The kinetics of 3HIBDH-IV for the degradation of 3-HP and several other organic acids were also examined using NAD^+^ as a cofactor. Ten different initial substrate concentrations were tested to measure the reaction rates. The apparent Michaelis-Menten constant was determined from the double reciprocal Lineweaver-Burk plots of the reaction rate vs. substrate and/or cofactor concentrations (Figure S1).

### Analytical Methods

The cell concentration was determined in a 10-mm-path-length cuvette using a double-beam spectrophotometer (Lambda 20, Perkin-Elmer, Norwalk, CT). One unit of absorbance at 600 nm corresponds to 0.3 g dried cell mass per liter. The protein concentrations in the cell-free extract were determined using the method described previously [16] on a microtiter plate reader (1420, Wallac Victor2; PerkinElmer) using bovine serum albumin as the standard.

### Homology Modeling of 3HIBDH-IV

The crystal structures of the human and bacterial 3HIBDHs templates (PDB ID: 2GF2, 2I9P, 2CKY, 3OBB, and 3Q3C) with high sequence identity and resolution were obtained using a BLAST search against the Protein Data Bank (PDB; www.rcsb.org) using the BLOSUM80 matrix with a gap penalty and gap extension penalty of 11 and 1, respectively. Table 2 provides details of the templates, which include the source, identity against target 3HIBDH-IV and their resolution. A model 3-dimentional (3D) structure of 3HIBDH-IV was created by comparative modeling using MODELLER 9v7 program. Briefly, the template structures were first aligned and superimposed. The resulting structures were used to align the target sequences of 3HIBDH-IV. A final alignment check and corrections were performed manually and 100 models were constructed using the MODELLER 9v7 program with high level optimization. The models were selected with the lowest probability density function (PDF) energy. The quality of the model was determined by examining the distribution of amino acid residues in the Ramachandran plot [17].

**Table 2:** Template sequences used for homology modeling of 3HIBDH-IV.

Target	Templates (PDB ID)	Source	Identity (%)	Resolution (A°)
	2GF2	Human	45.79	2.38
	2I9P	Human	46.10	2.55
3HIBDH-IV	3CKY	*Eubacterium barkeri*	41.33	2.30
	3OBB	*Pseudomonas aeruginosa*	50.51	2.20
	3Q3C	*Pseudomonas aeruginosa*	50.68	2.30

### Molecular Docking

A molecular docking study was carried out to examine the binding interaction between 3-HP and modeled 3HIBDH-IV. The AutoDock4.0 software package was used to run the docking protocol, as described previously [18]. The protein structure was prepared by assigning Kollman united-atom charges, solvation parameters and polar hydrogen atoms. The Gasteiger charge was added and the non-polar hydrogen atoms merged because the ligand molecule is not a peptide. The ligand molecule was kept flexible to rotate freely during the docking search. The active site residue of serine dehydrogenase, i.e. Lys171, which was reported previously in *Pseudomonas aeruginosa* (PDB ID: 3Q3C) [9], was considered to be the binding site for the modeled 3HIBDH-IV. The grid box was fixed at the center of the cavity site residue Lys171. The box size was 40×40×40 Å in the x, y and z axes, respectively. AutoGrid was used to produce grid maps. The space between grid points was 0.375 Å. The Lamarckian genetic algorithm (LGA) was used to search the conformer. Fifty docking runs were carried out by clustering analysis using the root-mean-square deviation (RMSD) of 2.0 Å with reference to the starting geometry of the ligand.

## Results

### RT-PCR Analysis of *3hibdh*s Expression in Wild Type *P. denitrificans* ATCC13867

**Figure 1:**
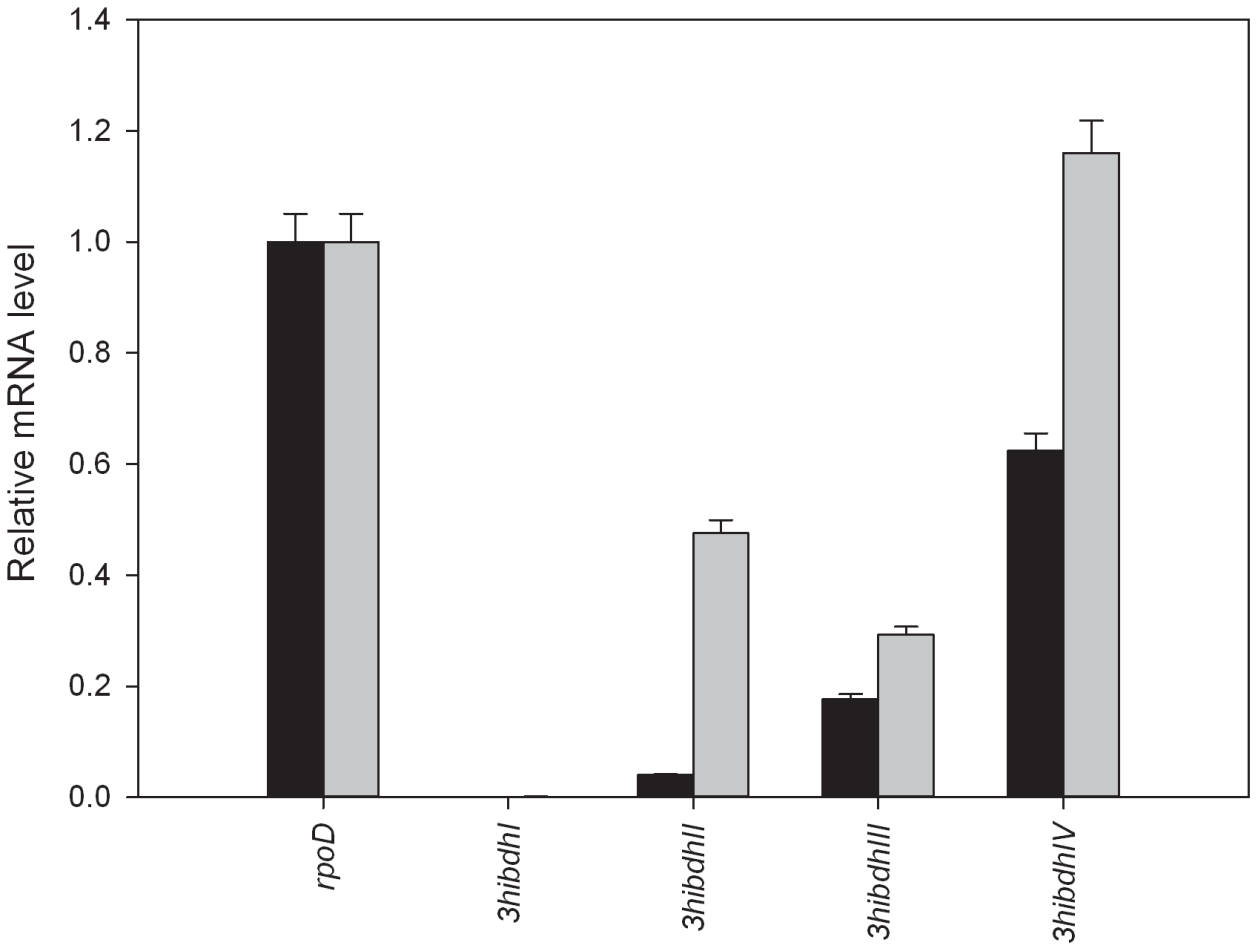
Analysis of relative mRNA levels for *3hibdhI, 3hibdhII, 3hibdhIII and 3hibdhIV* in wild type *P. denitrificans* ATCC13867. *P. denitrificans* ATCC13867 was cultured in M9 medium without (black bar) or with 3-HP supplementation (grey bar) at 30 mM and harvested in exponential growth period. The mRNA levels were estimated with *rpoD*, encoding sigma factor 70, as reference gene. The values are average of four experiments.

Figure 1 shows the mRNA expression level for *3hibdhI, 3hibdhII, 3hibdhIII and 3hibdhIV* in wild type *P. denitrificans* ATCC13867 cultured in M9 medium with and without 3-HP supplementation at 30 mM. The mRNA level of *rpoD* was constant regardless of 3-HP supplementation, suggesting that *rpoD* was a suitable housekeeping gene. The mRNA level for *3hibdhI* was negligible, while that for *3hibdhIV* was the highest among the four *3hibdh*s. The presence of 3-HP in the culture medium enhanced the expression of all *3hibdh* genes. The *3hibdhII* showed the highest improvement at 11.5±0.9-fold, followed by *3hibdhI* at 7.5±0.7-fold. In comparison, the improvement of mRNA level for *3hibdhIII* or *3hibdhIV* in the presence of 3-HP was less significant. However, despite the great improvement of expression levels, the mRNA levels for *3hibdhI* and *3hibdhII* were much lower than that for *3hibdhIV*. In a separate experiment, all four *3hibdh*s were introduced into *E. coli* and their activity in crude cell extract of the recombinant *E. coli* were measured (unpublished). The recombinant with *3hibdhIV* showed the highest activity among the four isozymes (3HIBDH-I, 2.88 U/mg protein; 3HIBDH-II, 2.85 U/mg protein; 3HIBDH-III, 0.00 U/mg protein; and 3HIBDH-IV, 3.52 U/mg protein). These results suggested that 3HIBDH-IV should be one of the major enzymes involved in 3-HP degradation, thus 3HIBDH-IV was chosen for further study.

### Sequence Analysis of 3HIBDH-IV and its Comparison with Other 3HIBDHs

**Figure 2:**
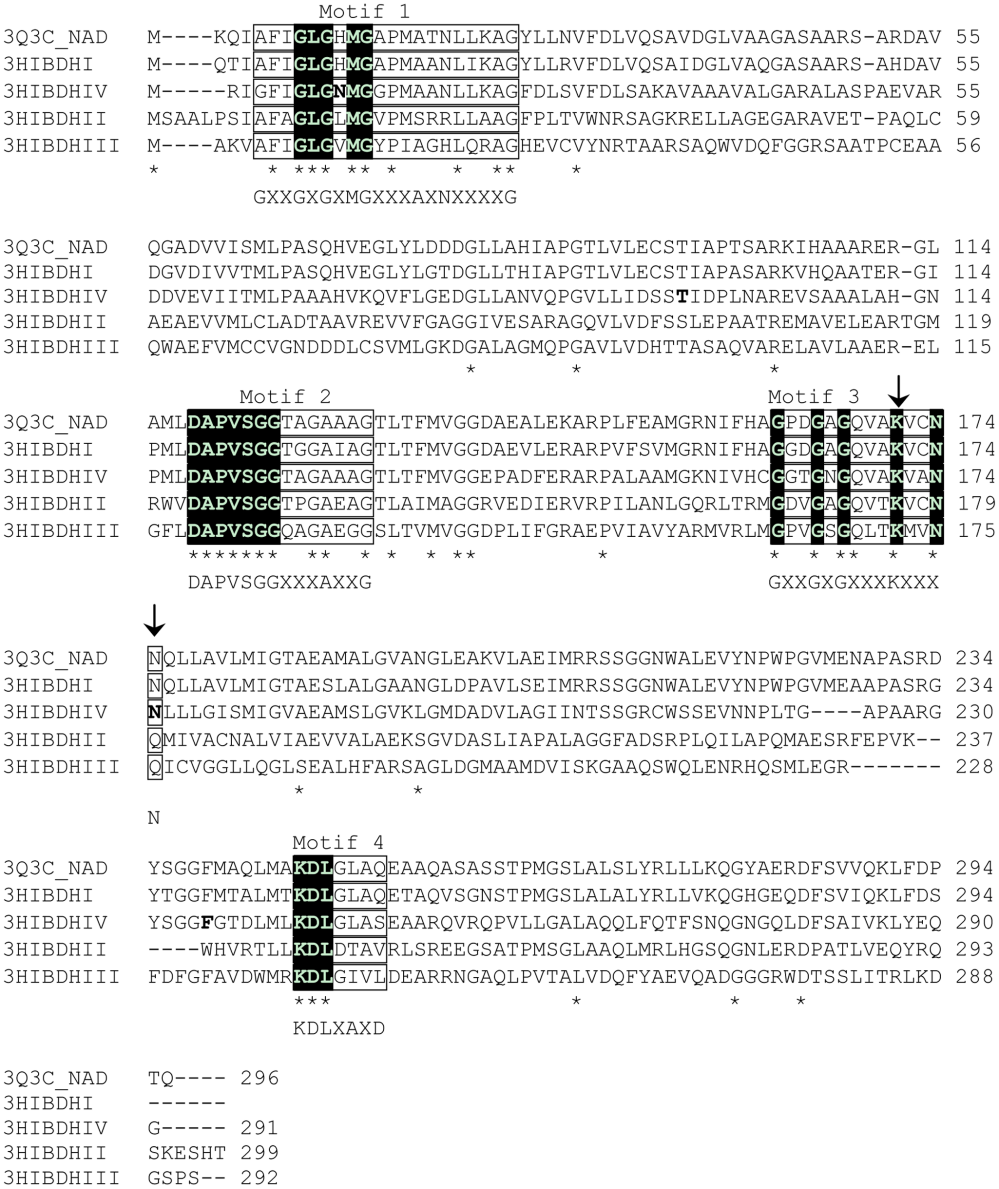
Amino acid sequence alignment of the putative 3-hydroxyisobutyrate dehydrogenase isozymes of *P. denitrificans* ATCC13867. The sequences of 3-hydroxyisobutyrate dehydrogenases (3HIBDH-I to IV) were compared with the structurally well-characterized _L_-serine dehydrogenase PA0743 (PDB ID: 3Q3C_NAD^+^) and the conserved invariant residues were highlighted in black with white letters. The putative active site common to the 3HIBDH family is indicated by the arrow marks. The sequences of the four most conserved motifs in the 3-hydroxyacid dehydrogenase family are boxed with the consensus sequences at the bottom. The conserved residues among the sequences are marked with a star.

The putative isozymes of 3-hydroxyisobutyrate dehydrogenases (3HIBDH-I to IV) were identified from the entire genome sequences of *P. denitrificans* ATCC 13867 using the Basic Local Alignment Search Tool (BLAST) search of the available amino acid sequence of 3HIBDH of *P. fluorescens* Pf5 ATCC BAA-477 (accession no., AAY90588) [10]. The amino acid sequences of the 3HIBDHs of *P. denitrificans* ATCC 13867 were compared with the structurally well-characterized _L_-serine dehydrogenase (PDB ID: 3Q3C_NAD) [9]. The deduced amino acids sequence of 3HIBDH-IV had 53.7% identity with 3Q3C_NAD, whereas that of 3HIBDH-I had 80.7% identity. Sequence analysis revealed the presence of four conserved motifs of the 3-hydroxyacid dehydrogenase family (Figure 2) [19]. Motifs 1 and 4 encode the cofactor binding site, and motifs 2 and 3 encode the substrate binding and catalysis sites, respectively. The residues in the N-terminal dinucleotide cofactor-binding motif 1 (GXXGXGXMGXXXAXNXXXXG) are 100% conserved in 3-hydroxyacid dehydrogenases [19]. This motif also determines the cofactor specificity (NAD^+^ or NADP^+^). The first seven residues in motif 2 (DAPVSGGXXXAXXG), substrate binding site were also highly conserved in the consensus sequences of the putative 3HIBDHs and 3Q3C_NAD^+^. Motif 3 (GXXGXGXXX**K**XXXN/Q) had highly conserved Lys171, which serves as the catalytic residue. In motif 4 (**K**DLGXAXD), which is located near the C-terminus, the first residue Lys240 was suggested to be surface exposed [19].

### Structure Analysis and Docking

**Figure 3:**
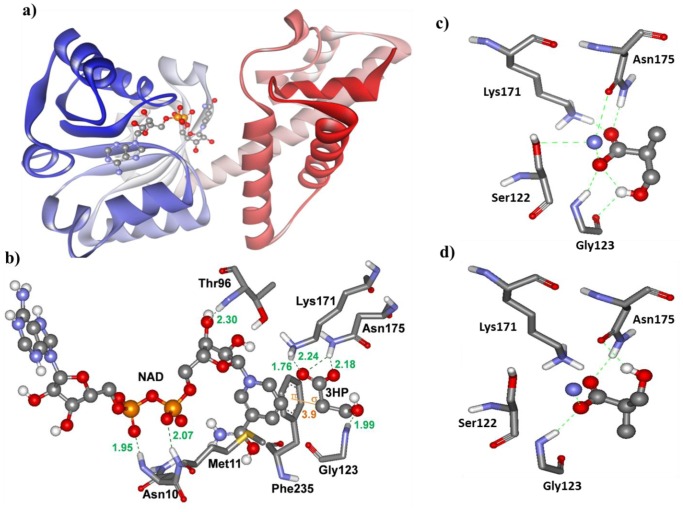
Predicted three dimensional (3D) structure of 3HIBDH-IV of *Pseudomonas denitrificans* ATCC 13867. The model shows the structure/interactions when the enzyme is complexed with NAD^+^ (**a**), NAD^+^ and 3-HP (**b**), (*S*)-3-hydroxyisobutyrate (**c**), and (*R*)-3-hydroxyisobutyrate (**d**). The interacting residues are represented with sticks and are labeled with residue numbers. The ligand molecules (NAD^+^, 3HP, (*S*)-3-hydroxyisobutyrate and (*R*)-3-hydroxyisobutyrate) are shown as scaled ball and sticks and are labeled. The hydrogen bonds and pi-sigma bonds are indicated as dotted lines and solid line, respectively, with the distance in Å. The colors represent different amino acids groups or atoms: blue, amino group; white, hydrogen atom; red, oxygen atom; grey, carbon chain; and orange, phosphorus.

A three-dimensional model of 3HIBDH-IV was developed to understand the structure and functional characteristics (Figure 3a). The model was generated using multiple templates along with NAD^+^ (Table 2). The predicted model was evaluated by calculating the main chain RMSD according to its high identity template structure (PDB ID: 3Q3C_A) and Ramachandran plot. The RMSD value of the predicted model was 0.77 Å, suggesting that the model prediction was satisfactory. Approximately 95% of the amino acids in the model shown in Figure 3a were distributed in the most favored regions of the Ramachandran plot, confirmed the reliability of the predicted model [17].

The docked simulation of 3-HP to the modeled 3HIBDH-IV produced a single cluster of conformers in the RMSD tolerance of 2.0 Å out of 50 runs of LGA. All conformations were clustered together and favored by producing 50 repeated poses, indicating the high binding specificity of 3-HP towards the binding site of 3HIBDH-IV. The lowest binding free energy complex of the first ranked cluster was used for analysis of the binding site residues. The protein-ligand complex (3HIBDH-IV –3-HP) was formed with multiple hydrogen bonds by the amino acid residues, Gly123, Lys171 and Asn175, and one pi-sigma interaction with the Phe235 aromatic ring (Figure 3b). A charged interaction between 3-HP and NAD^+^ located close to the substrate binding site was also detected. When (*S*)-3-hydroxyisobutyrate was docked with 3-HIBDH-IV, the Lys171, Asn175, Ser122 and Gly123 residues had a direct interaction (Figure 3c). On the other hand, Lys171 and Ser122 did not show any interaction when (*R*)-3-hydroxyisobutyrate was used (Figure 3d).

### Expression, Purification and Activity of Recombinant 3HIBDH-IV

**Figure 4:**
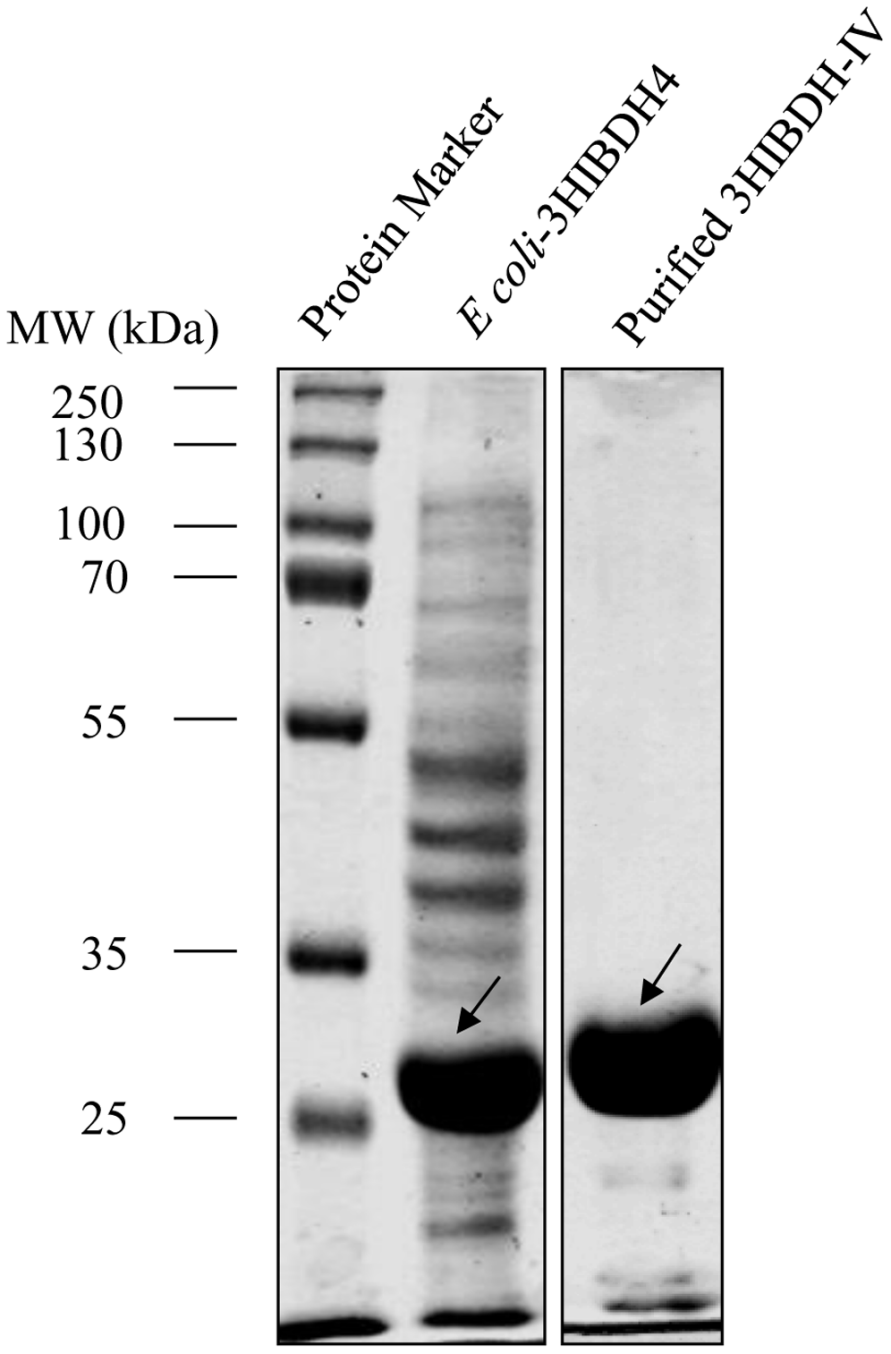
Expression and purification of recombinant 3HIBDH-IV protein by Ni-affinity column. 12% polyacrylamide gel was stained with Coomassie Brilliant Blue (G250).

Above sequence analysis and docking studies showed the possibility that 3-HP can be a substrate for 3HIBDH-IV. To confirm this, we purified the enzyme and investigated its activity on 3-HP. *E. coli* SH-3HIBDH4 was cultured in LB medium at 30°C and induced with 0.5 mM IPTG. SDS-PAGE analysis of the soluble crude cell lysate revealed the presence of the 3HIBDH-IV protein with an approximate molecular weight of 32 kDa (Figure 4, lane 2), which corresponds to the predicted size of 3HIBDH-IV. The target protein, as determined from the SDS-polyacrylamide gel image, was approximately 26% of the total cellular protein, and the ratio of the soluble and insoluble fractions of 3HIBDH-4 was 4∶1 (data not shown), which means approximately 80% of the targeted protein was expressed in a soluble form. His-tagged 3HIBDH-IV was purified to electrophoretic homogeneity using a Ni-NTA-HP resin column and the purified protein was observed as a single band on the SDS-polyacrylamide gel (Figure 4, lane 3).

Activity analysis of the soluble fraction from the crude cell lysate of SH-3HIBDH4 revealed a maximum specific activity of 3.36 U/mg protein with 3-HP as the substrate and NAD^+^ as the cofactor (Table 3). On the other hand, the specific activity of the purified 3HIBDH-IV was 16.95 U/mg protein for 3-HP with NAD^+^, showing 5-fold purity enrichment, as determined by the specific activity of 3HIBDH-IV. The protein yield estimated from the purified extract was approximately 26% of that of the crude cell extract.

**Table 3:** 3HIBDH-IV activities in the crude and purified enzyme extracts.

Enzyme	Protein (mg/mL)	Specific activity (U/mg protein)	Purification (fold)	Yield (%)
Crude extract	7.700±0.250	3.365±0.045	1	100
Purified 3HIBDH-IV	2.030±0.050	16.950±2.030	5.1	26

Assay conditions: 100 mM Tris-HCl buffer (pH 9.0); 3 mM 3-HP; 2 mM NAD^+^; and an appropriate amount of enzyme. The temperature was set at 30°C.

The values indicated are the triplicate measurement of the same enzyme preparation (mean values, n = 3).

The substrate specificity of 3HIBDH-IV was examined with various 3-hydroxyacids and amino acids in the presence of NAD^+^ as a cofactor. The purified 3HIBDH-IV had limited substrate specificity, and showed activity on C_4_ and C_3_ hydroxy acids, such as (*S*)-3-hydroxyisobutyrate, 3-hydroxypropionate and methyl-(*S*)-(+)-3-hydroxy-2-methylpropionate, and amino acids, such as _L_-serine and methyl-_D_,_L_-serine. On the other hand, it did not show any appreciable activity on (*R*)-3-hydroxyisobutyrate, methyl-2,2-dimethyl-3-hydroxypropionate and 3-hydroxybutyrate. 3-HP was the second most preferred substrate next to (*S*)-3-hydroxyisobutyrate for 3HIBDH-IV but its activity on 3-HP was 90% lower than that with (*S*)-3-hydroxyisobutyrate. 3HIBDH-IV could also convert _L_-serine, methyl-_D_,_L_-serine, and methyl-(*S*)-(+)-3-hydroxy-2-methylpropionate to their corresponding products, but less actively than 3-HP.

Kinetic studies showed that the half-saturation constant *K*_m_ of the purified 3HIBDH-IV for (*S*)-3-hydroxyisobutyrate was 0.178 mM (Table 4). The specific activity constant (*k*_cat_/*K*_m_) for (*S*)-3-hydroxyisobutyrate was 47 times higher than that for 3-HP, confirming that (*S*)-3-hydroxyisobutyrate is the natural substrate for 3HIBDH-IV in *P. denitrificans*. The maximum reaction rate for the oxidation of 3-HP to malonate semialdehyde was 20.243 U/mg protein using NAD^+^ as a cofactor. The *K_m_* for 3-HP was determined to be 1.055 mM, which is approximately 6 times higher than that for (*S*)-3-hydroxyisobutyrate but 16- and 82 times lower than that for 3-HIBDHs from *Bacillus cereus* and *P. putida* E23, respectively [7], [20].

**Table 4:** Kinetic properties of 3HIBDH- IV on the hydroxy acids and amino acids.

Substrates	*V*_max_ (U/mg protein)	*K*_m_ (mM)	k_cat_ (S^−1^)	*k*_cat_ */K*_m_×10^3^ (M^−1^S^−1^)
3-hydroxypropionate	20.243±1.772	1.055±0.035	10.800±0.945	10.228±0.699
Sodium (*S*)-3-hydroxyisobutyrate	151.434±2.069	0.178±0.010	80.790±1.103	454.861±33.021
L-serine	89.127±10.812	117.512±1.426	47.549±5.768	0.404±0.044
Methyl-D,L-serine	12.409±0.890	24.180±3.169	6.620±0.475	0.275±0.016
Methyl-(*S*)-(+)-3-hydroxy-2-methylpropionate	67.021±6.186	25.142±1.667	35.755±3.300	1.433±0.231
NAD^+a^	12.592±0.237	0.164±0.037	6.717±0.126	42.441±10.880

Assay conditions: 100 mM, Tris-HCl buffer (pH 9.0) containing 2 mM NAD^+^; 0.2 µg protein/mL; varied concentration of acids; temperature was set at 30°C.

a 3-HP was constant at 3 mM, and NAD^+^ varied between 0.025 and 4 mM.

The values indicated are the triplicate measurement of the same enzyme preparation (mean values, n = 3), except L-serine and Sodium (*S*)-3-hydroxyisobutyrate, for which an average of two independent values are indicated. Please refer to Supplementary data (Figure S1).

### Effect of Temperature and pH on 3HIBDH-IV Activity

**Figure 5:**
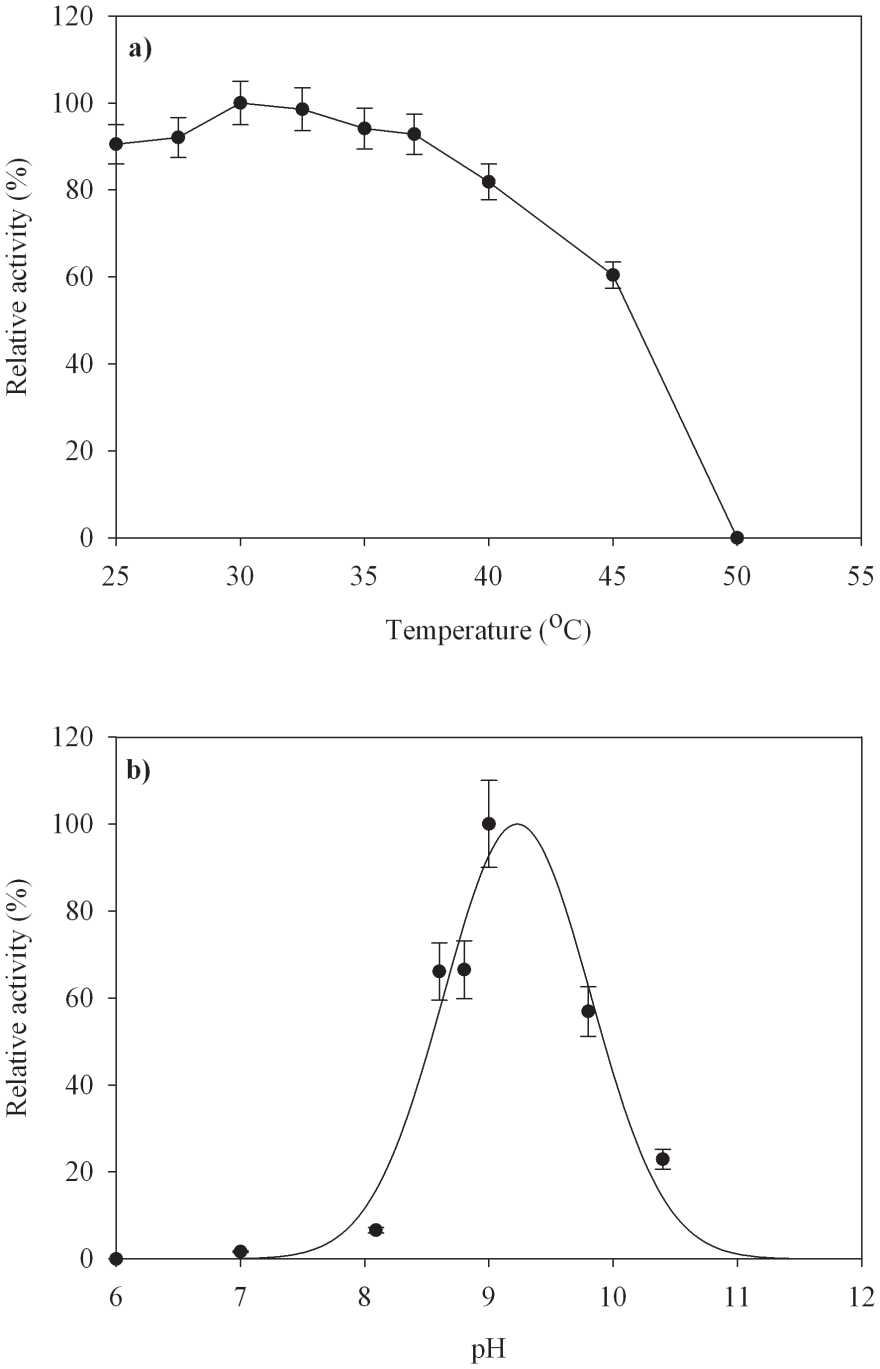
Effect of temperature and pH on the 3HIBDH-IV activity. (a) Relative activity at different temperatures; and (b) relative activity at different pH. Relative activity for pH and temperature are indicated as the mean values of triplicate measurement of the same enzyme preparation (n = 3). The relative values are expressed as a percentage of the values obtained at pH 9.0 or 30°C.

The effects of temperature on the activity of 3HIBDH-IV was examined from 25 to 50°C at pH 9.0 using 3-HP as a substrate (Figure 5a). The activity of 3HIBDH-IV of *P. denitrificans* was stable over a wide temperature range between 25 and 50°C. The effect of pH was examined in the range of 6.0 to 10.6 at 30°C (Figure 5b). The maximum activity was observed at pH 9.0 with more than 50% of the activity retained at pH 8.3 and 10.3. On the other hand, the activity was below the detectable level at pH ≤7.0.

### Effect of Metal Ions, Disulfide Reductant, and Other Compounds on 3HIBDH-IV Activity

The effects of different metal ions and chemical compounds on the 3HIBDH-IV activity were examined using 3-HP as a substrate and NAD^+^ as a cofactor at pH 9.0 and 30°C (Table 5). Most monovalent metal ions did not affect the 3HIBDH-IV activity. On the other hand, some transition metals, such as Cu^2+^, Zn^2+^ and Fe^2+^ inhibited the 3HIBDH-IV activity by more than 85%. In addition, Ag^+^ and Hg^2+^ inhibited 3HIBDH-IV completely. In contrast, the presence of the reducing agents, DTT and 2-ME, and the chelating agent, EDTA, increased its activity 1.5–1.8-fold.

**Table 5:** Effect of various metal ions and other chemical compounds on the 3HIBDH-IV activity with 3-HP as a substrate and NAD^+^ as a coenzyme.

Metal and other compounds^a^	Relative activity (%)
Positive monovalent light alkali metals	
NaCl	99.784±2.413
LiCl	101.680±2.343
NH_4_Cl	95.581±2.303
KCl	99.270±2.033
Positive bivalent alkali earth metal	
MgCl_2_	88.634±2.087
Transition metals	
CoCl_2_	64.321±1.877
NiSO_4_	62.247±1.787
CuSO_4_	14.519±0.489
ZnSO_4_	14.479±0.454
FeCl_2_	9.053±0.230
MnCl_2_	130.711±3.539
Heavy metals	
HgCl_2_	0.000
Ag_2_SO_4_	0.000
Disulfide reductants	
2-Mercaptoethanol	182.748±4.55
Dithiothreitol	184.827±4.922
Ethylenediaminetetraacetic acid	150.938±4.058

Assay conditions: 100 mM Tris-HCl buffer (pH 9.0); 3 mM 3-HP; 2 mM NAD^+^; and 0.2 µg protein/mL. The temperature was set at 30°C. 100% activity corresponds to 14.13 U mg^−1^ proteins.

a Added at 1mM.

The values indicated are the triplicate measurement of the same enzyme preparation (mean values, n = 3).

## Discussion

This study examined the 3-HP degrading enzyme in *P. denitrificans* ATCC 13867. 3HIBDH was selected as a target because this enzyme has been reported to catalyze the conversion of many 3-hydroxyacids, including 3-HP [8], . 3HIBDH-IV of *P. denitrificans* ATCC 13867 preferred NAD^+^ as a cofactor. No activity was detected with NADP^+^. 3HIBDH of a rabbit liver was reported to have a cofactor preference towards NAD^+^ [15]. Hawes et al. [21] suggested that the specific amino acid, either Asp or Arg, located after 6 hydrophobic residues from the final Gly residue of the consensus sequence “GXXGXGXMGXXXAXNXXXXG” in motif 1 (Figure 2), is important for determining the cofactor specificity. They also suggested that NAD^+^ is the preferred cofactor when Asp is present, whereas NADP^+^ is preferred when Arg is present [21]. The presence of Asp29 rather than Arg29 in 3HIBDH-IV of *P. denitrificans* ATCC 13867 agrees with this suggestion [21].

The 3HIBDH-IV activity exhibited strict enantio-selectivity towards (*S*)-3-hydroxyisobutyrate; it did not catalyze the oxidation of (*R*)-3-hydroxyisobutyrate. The docking results showed that the residues, Lys171, Asn175, Ser122 and Gly123, of 3HIBDH-IV had interactions with (*S*)-3-hydroxyisobutyrate (Figure 3c), whereas Lys171, an important catalytic residue, and Ser122 showed no interactions when docked with (*R*)-3-hydroxyisobutyrate (Figure 3d). This explains why 3-HIBDH-IV did not catalyze the oxidation of (*R*)-3-hydroxyisobutyrate. 3HIBDH of a rabbit liver was also reported to show a similar preference towards the *S*-enantiomer [15]. *S*-3-hydroxyisobutyrate is an intermediary metabolite of valine degradation, which connects the metabolisms of propionate, pyrimidine and citric acid. Therefore, it is expected that 3HIBDH plays an important role in these metabolisms, in addition to valine degradation. Few reports have shown that this enzyme can take 3-HP as an alternate substrate in the absence of its physiological substrate, 3-hydroxyisobutyrate [8], [21], [22], [23]. Docking experiments (Figure 3b) revealed four amino acids residues (Lys171, Asn175, Gly123 and Phe235) of 3HIBDH-IV to have direct interactions with 3-HP. In the case of_ L_-serine dehydrogenase PA0743 of *P. aeruginosa* PAO1, in addition to these four amino acids, Ser122 was suggested to play an important role in substrate binding [9], [23]. On the other hand, in 3-HIBDH-IV, Ser122 had no role when 3-HP was used as a substrate. Gly123 of substrate binding motif 2 (DAPVS**G**GXXXAXXG) had an interaction with 3-HP at the 3-hydroxyl group. Lys171 and Asn175 had direct interactions with the carboxylate group of 3-HP. Lys171 exhibited a charge interaction with the C−O^−^ group, whereas Asn175 formed hydrogen bonds with the C−O^−^ and C = O of the carboxylate group. The interaction of Lys171 and Asn175 with the carboxylate of serine for the catalytic activity has been reported in the crystal structure of serine dehydrogenase, PA0743 (PDB ID of PA0743−NAD^+^ complex: 3Q3C) [9], suggesting that 3HIBDH-IV shares a similar catalysis mechanism to the serine dehydrogenase PA0743 of *P. aeruginosa* PAO1. In addition, docking experiments enabled an estimation of the binding free energy for the reaction between the 3HIBDH-IV–NAD^+^ complex and 3-HP. The binding energy was −3.13 kcal/mol, suggesting that this binding is thermodynamically favorable.

The inhibitory effects on the 3HIBDH-IV activity of transition metals, such as Fe^2+^, Zn^2+^ and Cu^2+^, were much stronger than those of Co^2+^ and Ni^2+^, which is similar to that in rabbit liver 3HIBDH [15]. A complete loss of activity in the presence of Hg^2+^ and Ag^+^ is not unusual because these metals have strong affinity to thiol groups and form complexes with the sulfhydryl groups of sulfur containing amino acids, such as Cys and Met [24]. The docking result supports the importance of sulfur-containing amino acid residues, such as Met11 located in the NAD^+^ binding site. This suggests that Met11 is important for the function and/or integrity of the protein confirmation in 3HIBDH-IV.

One of the main objectives of this study was to characterize the 3-HP degradation activity of 3HIBDH-IV of *P. denitrificans* ATCC 13867. According to a resting-cell experiment, the 3-HP degradation activity of *P. denitrificans* was approximately 15 mmol/g cell/h at pH 7.0 (data not shown). In comparison, it was approximately 1,200 mmol/g protein/h with the purified 3HIBDH-IV at pH 9. However, the activity of purified 3HIBDH-IV was very sensitive to pH with almost no activity observed at pH 7.0. This suggests that the contribution of 3HIBDH-IV to 3-HP degradation in *P. denitrificans* ATCC 13867 can be insignificant at pH 7.0. To better understand the physiological significance of 3-HIBDH-IV, more study will be needed to disrupt the gene encoding 3HIBDH-IV from *P. denitrificans* ATCC 13867.

### Conclusion

The recombinant 3HIBDH-IV from *P. denitrificans* ATCC 13867 was cloned, expressed and characterized to understand its physicochemical properties. The purified 3HIBDH-IV was enantiospecific to (*S*)-3-hydroxyisobutyrate and preferred NAD^+^ as a cofactor for its catalytic reaction. 3-HP was a less preferred substrate than its physiological substrate (*S*)-3-hydroxyisobutyrate. Docking experiments revealed four amino acids residues, Lys171, Asn175, Gly123 and Phe235, to have direct interactions with 3-HP, and the binding between the enzyme–NAD^+^ complex and 3-HP was thermodynamically favorable (ΔG° = −3.13 kcal/mol). The 3-HP degradation activity of 3HIBDH-IV was quite sensitive to pH. The highest activity was observed at pH 9 but the activity became almost negligible at pH 7. Experiment involving the chromosomal deletion of 3*hibdhIV* in *P. denitrificans* to further elucidate its physiological role in 3-HP degradation and better understand 3HIBDH-IV are currently underway.

## Supporting Information

Figure S1 **Lineweaver-Burk plot derived from enzyme activity of 3HIBDH-IV on various substrates.**
(TIF)
