# Supplementary figures and images for: Cloning, Expression and Characterization of 3-Hydroxyisobutyrate Dehydrogenase from Pseudomonas denitrificans ATCC 13867
Source: PLoS One. 2013 May 1;8(5):e62666. doi: 10.1371/journal.pone.0062666 (PMC3642240; doi:10.1371/journal.pone.0062666)

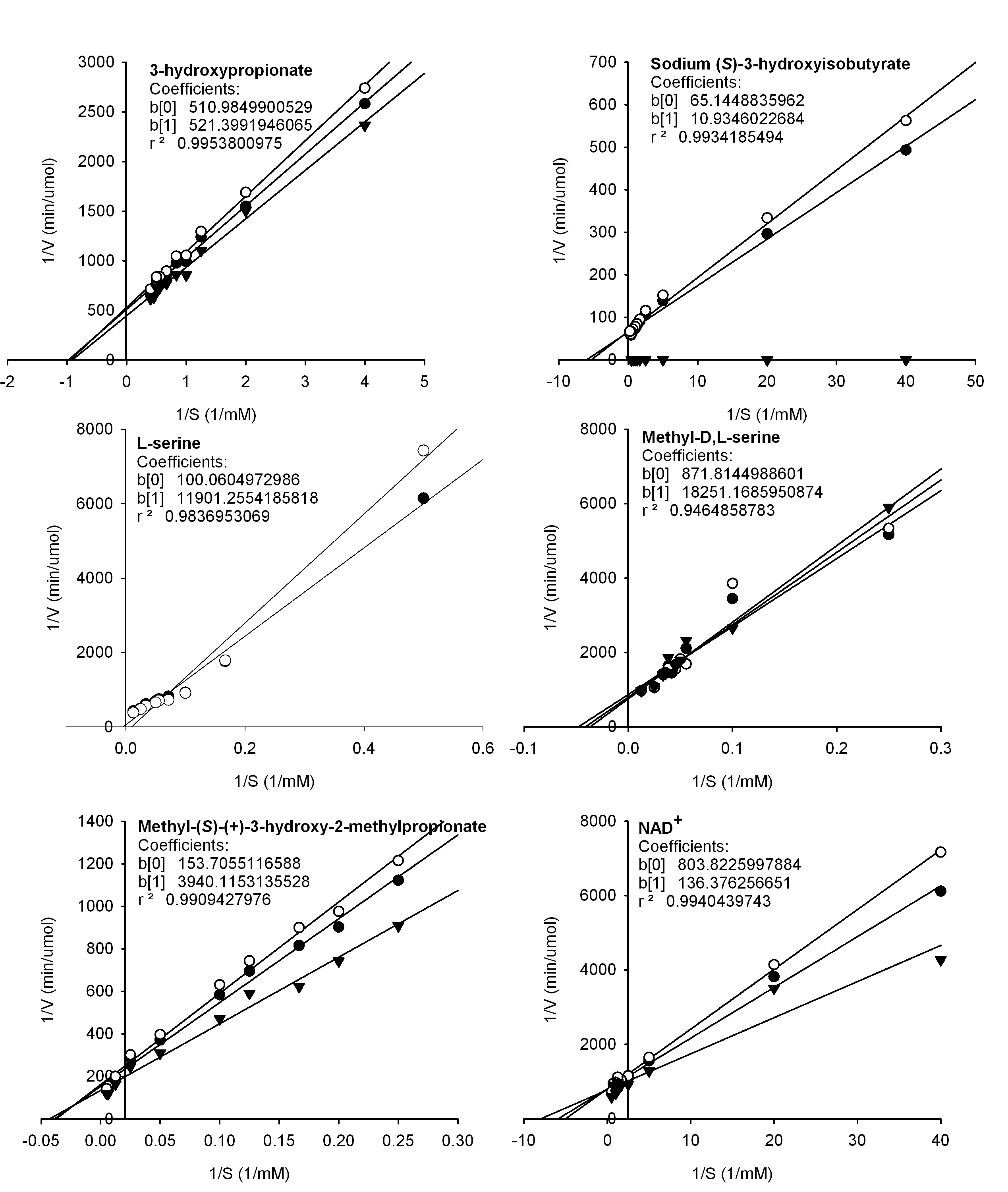

Supplement: Figure S1 — Lineweaver-Burk plot derived from enzyme activity of 3HIBDH-IV on various substrates. (TIF) [file pone.0062666.s001.tif]
